# Analyzing Association of the *XRCC3* Gene Polymorphism with Ovarian Cancer Risk

**DOI:** 10.1155/2014/648137

**Published:** 2014-06-10

**Authors:** Cunzhong Yuan, Xiaoyan Liu, Shi Yan, Cunfang Wang, Beihua Kong

**Affiliations:** ^1^Department of Obstetrics and Gynecology, Qilu Hospital of Shandong University, Jinan, Shandong 250012, China; ^2^Shandong Provincial Key Laboratory of Microbiological Engineering, Qilu University of Technology, Jinan, Shandong 250000, China

## Abstract

This meta-analysis aims to examine whether the *XRCC3* polymorphisms are associated with ovarian cancer risk. Eligible case-control studies were identified through search in PubMed. Pooled odds ratios (ORs) were appropriately derived from fixed effects models. We therefore performed a meta-analysis of 5,302 ovarian cancer cases and 8,075 controls from 4 published articles and 8 case-control studies for 3 SNPs of *XRCC3*. No statistically significant associations between *XRCC3* rs861539 polymorphisms and ovarian cancer risk were observed in any genetic models. For *XRCC3* rs1799794 polymorphisms, we observed a statistically significant correlation with ovarian cancer risk using the homozygote comparison (T2T2 versus T1T1: OR = 0.70, 95% CI = 0.54–0.90, *P* = 0.005), heterozygote comparison (T1T2 versus T1T1: OR = 1.10, 95% CI = 1.00–1.21, *P* = 0.04), and the recessive genetic model (T2T2 versus T1T1+T1T2: OR = 0.67, 95% CI = 0.52–0.87, *P* = 0.002). For *XRCC3* rs1799796 polymorphisms, we also observed a statistically significant correlation with ovarian cancer risk using the heterozygote comparison (T1T2 versus T1T1: OR = 0.91, 95% CI = 0.83–0.99, *P* = 0.04). In conclusion, this meta-analysis shows that the *XRCC3* were associated with ovarian cancer risk overall for Caucasians. Asian and African populations should be further studied.

## 1. Introduction

Ovarian cancer is the leading cause of the female reproductive system, with over 220,000 new cases and over 140,000 deaths worldwide in 2008 [1]. As most of the carcinomas, ovarian cancer is a multifactorial disease. Genetic factors are considered to influence the susceptibility of glioma genetic factors which all play significant roles in its susceptibility [2]. The genetic basis of ovarian carcinogenesis has been investigated in many studies.* BRCA1*,* BRCA2*,* MLH1*,* MSH2*,* SMAD6*,* RAD51C*,* RAD51D*,* RB1*,* LIN28B*,* CASP8*, and* MTDH* have all been implicated [3–11]. Recently, several common susceptibility alleles in four loci to be strongly associated with ovarian cancer risk have been found in three genome-wide association studies (GWAS) [12–14]. Examination of gene polymorphisms may explain individual differences in cancer risk [15].

*XRCC3* (X-ray repair cross-complementing group 3) belongs to a family of genes responsible for repairing DNA double strand breaks caused by normal metabolic processes or exposure to ionizing radiation [16].* XRCC3* interacts and stabilizes Rad51 and involves in HRR (homologous recombinational repair) for DBSs (double strand breaks of DNA) and cross-link repair in mammalian cells [17, 18]. The SNP rs861539 lead to Thr241Met amino acid substitution, that may affect the function and/or its interaction with other proteins involved in DNA damage and repair [17, 19]. The SNP rs1799794 (4541* A* >* G*) is located in 5′UTR and the SNP rs1799796 (17893* A* >* G*) is located in intron 5 [20]. So the two SNPs do not change the proteins of* XRCC3*.* XRCC3* polymorphism was associated with the risks of many cancers, such as lung cancer, breast cancer, and head and neck cancer [21–24]. The association between* XRCC3* polymorphism and ovarian cancer has been studied [20, 25–29]; however, those experimental results remain confusing. To summarize the effect of the* XRCC3* polymorphism on the risk for ovarian cancer, we performed a meta-analysis.

## 2. Methods

### 2.1. Search and Selection Process

The search of the PubMed database was performed using the following keywords: “X-ray repair cross-complementing group 3,” “*XRCC3*,” “rs861539,” “T241M,” “rs1799794,” “a4541g,” “rs1799796,” “a17893g,” “polymorphism,” “ovarian cancer,” and their combination. Two authors (Yuan and Wang) independently checked all the references retrieved to assess their appropriateness for the inclusion in this meta-analysis. In addition, we checked all the references cited in the articles and relevant reviews. For overlapping and republished studies, only the study with the largest samples was included. If an article reported results including different studies, each study was treated as a separate comparison in our meta-analysis.

Included studies met 3 criteria:

1. evaluating the association between* XRCC3* polymorphisms and ovarian cancer risk;
2. using sufficient published data to enable estimation of an odds ratio (OR) with its 95% confidence interval (CI);
3. using respective or prospective cohort case-control studies.

### 2.2. Data Extraction

Two authors (Yuan and Wang) independently extracted data from selected articles according to the inclusion criteria and reached a consensus on all items.

The following information was extracted from each study if available: the first author, year of publication, countries, area of the cases, the ethnicity of the population, the cases source, the sample type of cases, the numbers of cases and controls, and the genotype distributions of* XRCC3* in both cases and controls.

### 2.3. Quality Score Assessment

Two authors independently evaluated the quality of the 8 studies according to the scale for quality assessment (Table 1), which has been described previously [30, 31]. Quality score assessment was performed according to “source of cases,” “source of controls,” “specimens of cases for determining genotypes,” “Hardy-Weinberg equilibrium in controls,” and “total sample size.” Total scores ranged from 0 (worst) to 15 (best). Studies scoring ≥10 were defined as “high quality,” and those <10 were defined as “low quality.”

### 2.4. Statistical Analysis

Pooled ORs with 95% CIs were calculated to access the strength of association between* XRCC3* polymorphism and ovarian cancer susceptibility, according to the genotype frequencies of cases and controls groups [32]. *P* < 0.05 was considered statistically significant; all tests and CIs were two sided. If the heterogeneity was significant, the pooled ORs were initially measured by the random effects model. Else, the fixed-effects model was chosen [33].

The* XRCC3* polymorphism and ovarian cancer risk were performed for a homozygote comparison (T2T2 versus T1T1), heterozygote comparison (T1T2 versus T1T1), dominant genetic model (T1T2+T2T2 versus T1T1), and the recessive genetic model (T2T2 versus T1T1+T1T2). In addition, sensitivity analysis was performed by omitting each study. Publication bias was estimated using a funnel plot. The degree of asymmetry was examined by t Egger's test (*P* < 0.05 was considered significant publication bias) [34]. The analysis was carried out using Review Manager statistical software (RevMan version 5.0.17.0; The Nordic Cochrane Center, Rigshospitalet, Copenhagen, Denmark) and STATA software (version 11.2, Stata Corporation, College Station, TX, USA). Hardy-Weinberg equilibrium (HWE) was calculated using a web-based statistical tool (http://ihg.gsf.de/cgi-bin/hw/hwa1.pl).

## 3. Results

### 3.1. Study Characteristics

**Figure 1:**
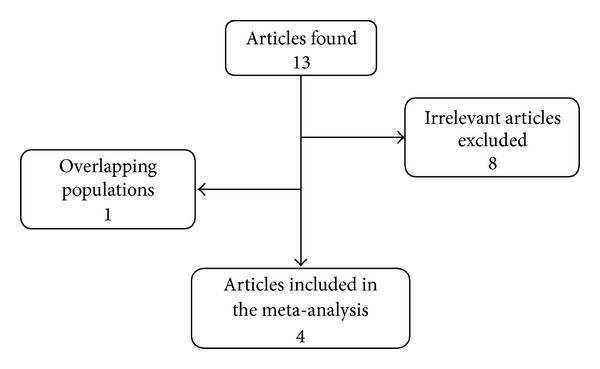
Study flow chart explaining the selection of the four articles included in the meta-analysis.

Through the literature search, 13 articles were found. Eight articles [35–42] were excluded as irrelevant study. One study [26] was excluded because it was carried out on overlapping populations with another, more samples eligible study [27]. Total 4 articles including 8 studies were selected on 5,302 ovarian cancer cases and 8,075 controls for 3 SNPs [20, 25–27] (Figure 1). These studies were all published in English. The main characteristics of the 4 studies are shown in Table 2. All subjects in these studies were Caucasians. The sample sizes (cases and controls) ranged from 1,478 to 5,906. Quality scores for all studies were high quality (≥10). Distribution of rs861539 polymorphisms genotype frequencies among ovarian cancer cases and controls of the 2 studies is shown in Table 3. Distribution of rs1799794 polymorphisms genotype frequencies is shown in Table 4 and distribution of rs1799796 polymorphisms genotype frequencies is shown in Table 5.

Hardy-Weinberg disequilibrium of genotype frequencies among the controls was calculated in three studies.

### 3.2. Association of Individual Polymorphisms with Ovarian Cancer

The heterogeneity analysis has been carried out. As it was shown in Tables 3, 4, and 5, the heterogeneities of 3 SNPs are all not significant. So the fixed-effects model was chosen for 3 SNPs.

The meta-analysis results of* XRCC3* rs861539 polymorphisms are shown in Table 3. No statistically significant associations between* XRCC3* rs861539 polymorphisms and ovarian cancer risk were observed in any genetic models (T2T2 versus T1T1: OR = 0.95, 95% CI = 0.85–1.06, *P* = 0.37; T1T2 versus T1T1: OR = 0.95, 95% CI = 0.88–1.03, *P* = 0.22; T1T2+T2T2 versus T1T1: OR = 0.95, 95% CI = 0.88–1.02, *P* = 0.19; T2T2 versus T1T1+T1T2: OR = 0.97, 95% CI = 0.88–1.08, *P* = 0.63).

For* XRCC3* rs1799794 polymorphisms, two studies [16, 18, 20, 21, 23, 24] (3,119 cases and 6,207 controls) were eligible. The meta-analysis results of rs1799794 polymorphisms are shown in Table 4. We observed a statistically significant correlation with ovarian cancer risk using the homozygote comparison (T2T2 versus T1T1: OR = 0.70, 95% CI = 0.54–0.90, *P* = 0.005), heterozygote comparison (T1T2 versus T1T1: OR = 1.10, 95% CI = 1.00–1.21, *P* = 0.04), and the recessive genetic model (T2T2 versus T1T1+T1T2 : OR = 0.67, 95% CI = 0.52–0.87, *P* = 0.002). However, no statistically significant associations were observed in dominant genetic model (T1T2+T2T2 versus T1T1: OR = 1.06, 95% CI = 0.96–1.15, *P* = 0.24).

For* XRCC3* rs1799796 polymorphisms, the meta-analysis results were shown in Table 4. We observed a statistically significant correlation with ovarian cancer risk using the heterozygote comparison (T1T2 versus T1T1: OR = 0.91, 95% CI = 0.83–0.99, *P* = 0.04). However no statistically significant associations were observed in homozygote comparison (T2T2 versus T1T1: OR = 1.07, 95% CI = 0.93–1.24, *P* = 0.33), dominant genetic model (T1T2+T2T2 versus T1T1: OR = 0.94, 95% CI = 0.86–1.03, *P* = 0.16), and the recessive genetic model (T2T2 versus T1T1+T1T2: OR = 1.13, 95% CI = 0.98–1.29, *P* = 0.08).

### 3.3. Publication Bias and Sensitivity Analysis

The publication bias was tested by Begg's funnel plot and Egger's test for three SNPs. Egger's test results did not show any evidence of publication bias for any of the genetic models of the three SNPs (data not shown). The shape of the four Begg's funnel plots showed no evidence of obvious asymmetry of the three SNPs (data not shown).

In the sensitivity analysis, the corresponding pooled ORs were not altered, when the fixed-effects model was changed to random-effects model. So it revealed that the results of this meta-analysis were stable.

## 4. Discussion

The* XRCC3* gene is required for genomic stability [36]. It was reported that the* XRCC3* polymorphism increased the risk of many cancers, including ovarian cancer [36]. However, the results have been inconsistent. We preformed the meta-analysis including 5,302 ovarian cancer cases and 8,075 controls for 3 SNPs of* XRCC3*.

For rs861539 polymorphisms, no correlation with ovarian cancer risk was observed in any genetic models. However, For* XRCC3* rs1799794 and rs1799796 polymorphisms, we observed a statistically significant correlation with ovarian cancer risk. It was shown that the difference between different SNP sites was considerable for* XRCC3*.

All of the literature was of high quality. All study subjects were Caucasian. The global multicenter studies can provide more valuable conclusions. So further studies should be done to explore the possible relationships between* XRCC3* polymorphisms and ovarian cancer risk in other ethnicities.

In conclusion, this meta-analysis shows that the* XRCC3* were associated with ovarian cancer risk overall for Caucasians. Asian and African populations should be further studied.

## Figures and Tables

**Table 1:** Scale for quality assessment.

Criteria	Score
Source of cases	
Population or cancer registry	3
Mixed (hospital and cancer registry)	2
Hospital	1
Other	0
Source of controls	
Population based	3
Volunteers or Blood bank	2
Hospital based (cancer-free patients)	1
Not described	0
Specimens of cases for determining genotypes	
Blood or normal tissues	3
Mixed (blood and archival paraffin blocks)	1
Tumor tissues or exfoliated cells of tissue	0
Hardy-Weinberg equilibrium in controls	
Hardy-Weinberg equilibrium	3
Hardy-Weinberg disequilibrium	0
Total sample size	
≥1000	3
≥500 and <1000	2
≥200 and <500	1
<200	0

**Table 2:** Main characteristics of the studies included in the meta-analysis.

First author	Year	Country	Area of the cases	Ethnicity	Cases source	Controls source	Sample type of cases	Total cases/controls	Quality score
Auranen [20]	2005	Mixed (UK-USA)	Royal Marsden Hospital in London and 6 counties in Northern California	Caucasian	Mixed (hospital and cancer registry)	Population	Blood	1665/4241	14

Beesley [26]	2007	Australia	New South Wales and Victorian Cancer Registries	Caucasian	Cancer registry	Population	Blood	731/747	15

Quaye [25]	2009	Mixed (DK-UK-USA)	MALOVA from Denmark-SEARCH from the UK-and GEOCS from the USA.	Caucasian	Mixed (hospital and cancer registry)	Population	Blood	1461/2299	14

Webb [27]	2005	Australia	New South Wales-Victoria and Queensland	Caucasian	Mixed (hospital and cancer registry)	Volunteers	Mixed (blood and archival paraffin blocks)	1445/788	12

**Table 3:** Distribution of *XRCC3* rs861539 genotype among ovarian cancer cases and controls included in the meta-analysis.

First author	Year	Genotypes distribution (Case source)			Genotypes distribution (Controls source)			P-HWE (Controls)	T2T2 versus T1T1		T1T2 versus T1T1		T1T2+T2T2 versus T1T1		T2T2 versus T1T1+T1T2	
		T1T1	T1T2	T2T2	T1T1	T1T2	T2T2		OR (95% CI)	*P*	OR (95% CI)	*P*	OR (95% CI)	*P*	OR (95% CI)	*P*
Auranen [20]	2005	676	762	227	1712	1946	583	Yes	0.99 [0.83, 1.18]	0.88	0.99 [0.88, 1.12]	0.89	0.99 [0.88, 1.11]	0.87	0.99 [0.84, 1.17]	0.91
Beesley [26]	2007	291	339	101	288	351	108	Yes	0.93 [0.67, 1.27]	0.63	0.96 [0.77, 1.19]	0.69	0.95 [0.77, 1.17]	0.62	0.95 [0.71, 1.27]	0.72
Quaye [25]	2009	545	612	175	784	958	282	Yes	0.89 [0.72, 1.11]	0.31	0.92 [0.79, 1.07]	0.27	0.91 [0.79, 1.05]	0.21	0.93 [0.76, 1.14]	0.51
Webb [27]	2005	591	656	198	307	375	106	Yes	0.97 [0.74, 1.28]	0.83	0.91 [0.75, 1.10]	0.32	0.92 [0.77, 1.10]	0.37	1.02 [0.79, 1.32]	0.87

Total		2103	2369	701	3091	3630	1079		0.95 [0.85, 1.06]	0.37	0.95 [0.88, 1.03]	0.22	0.95 [0.88, 1.02]	0.19	0.97 [0.88, 1.08]	0.63
									**Test for ** **heterogeneity**	*P* = 0.91	**Test for** **heterogeneity**	*P* = 0.88	**Test for** **heterogeneity**	*P* = 0.82	**Test for** ** heterogeneity**	*P* = 0.77

**Table 4:** Distribution of *XRCC3* rs1799794 genotype among ovarian cancer cases and controls included in the meta-analysis.

First author	Year	Genotypes distribution (Case source)			Genotypes distribution (Controls source)			P-HWE (Controls)	T2T2 versus T1T1		T1T2 versus T1T1		T1T2+T2T2 versus T1T1		T2T2 versus T1T1+T1T2	
		T1T1	T1T2	T2T2	T1T1	T1T2	T2T2		OR (95% CI)	*P*	OR (95% CI)	*P*	OR (95% CI)	*P*	OR (95% CI)	*P*
Auranen [20]	2005	1060	550	48	2551	1188	161	Yes	0.72 [0.52, 1.00]	0.048	1.11 [0.98, 1.26]	0.087	1.07 [0.95, 1.20]	0.29	0.69 [0.50, 0.96]	0.027
Quaye [25]	2009	940	484	37	1505	713	89	Yes	0.67 [0.45, 0.99]	**0.04**	1.09 [0.94, 1.25]	0.25	1.04 [0.91, 1.19]	0.57	0.65 [0.44, 0.96]	0.027

Total		2000	1034	85	4056	1901	250		0.70 [0.54, 0.90]	0.005	1.10 [1.00, 1.21]	0.04	1.06 [0.96, 1.15]	0.24	0.67 [0.52, 0.87]	0.002
									**Test for ** **heterogeneity**	*P* = 0.77	**Test for** **heterogeneity**	*P* = 0.83	**Test for** **heterogeneity**	*P* = 0.78	**Test for** **heterogeneity**	*P* = 0.80

**Table 5:** Distribution of *XRCC3* rs1799796 genotype among ovarian cancer cases and controls included in the meta-analysis.

First author	Year	Genotypes distribution (Case source)			Genotypes distribution (Controls source)			P-HWE (Controls)	T2T2 versus T1T1		T1T2 versus T1T1		T1T2+T2T2 versus T1T1		T2T2 versus T1T1+T1T2	
		T1T1	T1T2	T2T2	T1T1	T1T2	T2T2		OR (95% CI)	*P*	OR (95% CI)	*P*	OR (95% CI)	*P*	OR (95% CI)	*P*
Auranen [20]	2005	769	692	203	1757	1776	433	Yes	1.07 [0.89, 1.29]	0.47	0.89 [0.79, 1.01]	0.062	0.93 [0.83, 1.04]	0.188	1.13 [0.95, 1.35]	0.17
Quaye [25]	2009	676	608	177	1040	1006	253	Yes	1.08 [0.87, 1.33]	0.5	0.93 [0.81, 1.07]	0.31	0.96 [0.84, 1.09]	0.536	1.11 [0.91, 1.37]	0.30

Total		1445	1300	380	2797	2782	686		1.07 [0.93, 1.24]	0.33	0.91 [0.83, 0.99]	0.04	0.94 [0.86, 1.03]	0.16	1.13 [0.98, 1.29]	0.08
									**Test for ** **heterogeneity**	*P* = 0.97	**Test for ** **heterogeneity**	*P* = 0.65	**Test for ** **heterogeneity**	*P* = 0.69	**Test for ** **heterogeneity**	*P* = 0.90

## Abbreviations

CIs: Confidence intervals
HWE: Hardy-Weinberg equilibrium
ORs: Odds ratios
*XRCC3*: X-ray repair cross-complementing group 3.
